# The influences of DNA methylation and epigenetic clocks, on metabolic disease, in middle-aged Koreans

**DOI:** 10.1186/s13148-020-00936-z

**Published:** 2020-10-15

**Authors:** Ho-Sun Lee, Taesung Park

**Affiliations:** 1 Interdisciplinary Program in Bioinformatics and Department of Statistics, Seoul National University, 1 Kwanak-Ro, Kwanak-gu, Seoul, 151-747 Republic of Korea; 2 Toxicology Division, Daegu Institute, National Forensic Service, 33-14 Hogukrp, Waegwaneup, Gyeongsangbukdo, 39872 Republic of Korea

**Keywords:** Metabolic syndrome, Epigenetic clock, DNA methylation age, GrimAge

## Abstract

**Background:**

Considering that DNA methylation (DNAm) profiles are, in large part, modifiable by lifestyle and environmental influences, it has been proposed that epigenetic clocks provide a better estimate of biological age than chronological age, as associated with current health status. Even though metabolic diseases induce precocious aging, little is known about associations between metabolic syndrome (MetS) and DNA methylation clocks, and stochastic epigenetic mutations (SEMs), in a Korean population. Therefore, we assessed four different epigenetic clocks (Pan-tissue, Hannum, PhenoAge, and GrimAge), and their accelerations, on MetS and MetS-related lifestyle factors, in Koreans. We measured genome-wide DNA methylation (485,512 CpGs), using an Illumina 450 methylation BeadChip array, with data from 349 blood samples.

**Results:**

DNAm GrimAge strongly correlated with chronological age (*r* = 0.77, *p* < 0.001) compared to the other three epigenetic clocks and SEMs. DNAm-based surrogate markers, with regard to MetS, including the gene encoding plasminogen activator inhibitor-1 (PAI1), also correlated with chronological age. Within cohorts stratified by age group, sex, regional area, smoking, and alcohol drinking, a positive correlation was observed between DNAm GrimAge and chronological age (0.43 ≤ *r* ≤ 0.78). In particular, we identified MetS to associate with accelerated GrimAge, and age-adjusted PAI1, in the middle-age group. Accerelated GrimAge also associated with risk of MetS in the middle-age group (odds ratio = 1.16, *p* = 0.046), which appears to mediate their associations with fasting glucose. Multiple linear regression showed that DNAm GrimAge, and its acceleration, associate with MetS scores, in the middle-age group (*r* = 0.26, *p =* 0.006). Age-adjusted PAI1 was also significantly different between the MetS and control groups, and further associated with MetS scores (*r* = 0.31, *P* < 0.001), in the middle age group.

**Conclusion:**

DNAm GrimAge is a surrogate marker for MetS, and its component score, in Koreans. This association can be observed only in middle age. Therefore, appropriate DNA methylation clocks may aid in the prediction of Korean metabolic diseases.

## Background

Elderly age is growing dramatically faster than any other age group around the world. According to the United Nations Population Division, approximately 900 million people are 60 years or older worldwide, accounting for 21% of the global population by 2050 [1]. Therefore, understanding the biological process of aging could help promote healthy aging, longevity, and the prevention of age-related chronic diseases. Aging is the most universal contributor to the etiology of metabolic diseases, due to changes in energy regulation, and a progressive decline in functional integrity and homeostasis, culminating in death [2]. From biochemical, pathophysiological, and hormonal standpoints, metabolic syndrome (MetS) can be considered a sign of rapid aging that determines age-related metabolic features. For example, the common individual components of MetS, in elderly persons, are hypertension, glucose abnormalities, and central obesity [3].

One theory of aging involves the production of free radicals that oxidatively modify cellular constituents, resulting in mitochondrial dysfunction and the loss of cellular homeostasis, during biological aging [4]. Other theories suggest that epigenetic alterations play a huge role in the aging process [5, 6]. It has been noted that genome-wide DNA methylation levels decline with age. However, the significance of this change remained unknown until it became possible to measure the methylation status, of specific genomic sites. It was observed that while the methylation of some sites does indeed decrease with age, that of others increase or remain unchanged.

The application of machine learning methods to quantify DNAm changes, in multiple sites, allowed the generation of a highly accurate estimator of age, called the epigenetic “clock.” DNA methylation, based on a set of CpG dinucleotides, in specific cell types, creates a DNA methylation clock (referred to as epigenetic age), which reflects cellular age [7–9]. The widely accepted epigenetic clocks of Horvath [8] and Hannum [7] are quite accurate, with correlation coefficients > 0.9, with chronological age. Recently, the DNA methylation predictor (DNAm) GrimAge was reported to accurately predict time to death, and time to the onset of many human diseases, including cancer and heart disease [10]. Interestingly, epigenetic age acceleration (deviation between chronological age and epigenetic age) is now considered a biomarker of aging, predictive of premature morbidity, and mortality. Moreover, stochastic epigenetic mutations (SEMs) in DNA methylation occur, over the lifespan, due to slight imprecision of the epigenetic maintenance machinery, with particular regard to cardiovascular pathology and human aging [11, 12].

The application of epigenetic clocks to large human epidemiological data sets revealed that discordance between predicted (epigenetic age) and chronological age associated with many age-related pathologies, particularly when epigenetic age is greater than chronological age. Nannini et al. reported that MetS associates with intrinsic and extrinsic epigenetic age acceleration, in young adults [13]. However, there are few studies of MetS and epigenetic age, and the majority of these studies comprised populations of Caucasian ancestry. Therefore, it is necessary to study the role of DNA methylation, in the biology of age-related disparities, among ethnic minorities.

The objectives of this study were to assess the possible association of epigenetic age and SEMs with MetS, using lifestyle factors such as physical and blood traits, in a Korean population. In addition, we investigated the association between accelerated DNAm age and MetS, along with its pathological components. We also divided chronological age into middle-age and elderly groups, to investigate which group is more suitable for predicting age-related metabolic conditions, using DNAm age.

## Results

### Characteristics

The baseline characteristics for MetS cases and controls are summarized in Table 1. The participants consisted of 349 individuals (women *n* = 172, men *n* = 177), with a chronological age of 60.72 (range, 47–77 years). Mean chronological age for MetS controls was 59.74 years, whereas the mean chronological age for MetS was 62.25 years. 49.3% of participants in this study were females. 22.1% and 47.1% of MetS patients were current smokers, and current alcohol drinkers, respectively. MetS cases were more likely to be urban inhabitants and have lower educational levels and lower income. We also found no significant differences in smoking status, alcohol consumption, or physical activity, between the MetS and control groups. When calculated across four epigenetic clocks (Pan-tissue, Hannum, PhenoAge, and GrimAge) and SEMs, in the MetS state, DNAm GrimAge in MetS was significantly higher than those of the controls (65.05 years for control; 67.32 years for MetS, *p* =0.004 (Table 2)). In methylation-based surrogates of plasma proteins and smoking pack-years, PAI1 (18346.47 for control and 19880.95 for MetS, *p* < 0.001) and TIMP1 (34309.52 for control and 34686.02 for MetS, *p* =0.004), in MetS, respectively, were significantly higher than controls. Characteristics between the middle-aged and elderly groups are presented in Supplementary Table 1.

**Table 1:** Characteristics of the study subjects

	Control (*N* = 213)	MetS (*N* = 136)	*p* value
Female (%)	108 (62.8)	64 (37.2)	0.579
Chronological age (years)	59.74 (8.21)	62.25 (8.49)	< 0.007
Living area			<0.078
Urban	59	140	
Rural	104	46	
Smoke			0.636
Never smoker	134 (62.9)	83 (61)	
Former smoker	37 (17.4)	23 (16.9)	
Current smoker	42 (19.7)	30 (22.1)	
Alcohol			
Never drinker	106 (49.8)	62 (45.6)	0.824
Non-regular	3 (1.4)	10 (7.4)	
Regular drinker	104 (48.8)	64 (47.1)	
Education			0.155
Elementary school or less	22	91	
Middle school graduate	13	22	
High school or higher	24	27	
Income (USD)			0.011
< 1000	19	115	
1000–2000	29	27	
≥ 2000	114	38	
Physical activity (MET/day)	90.40 (13.84)	91.49 (13.21)	0.555
Waist (cm)	80.34 (7.77)	91.99 (6.98)	< 0.001
Hip (cm)	89.25 (4.92)	94.31 (5.50)	< 0.001
WHR	0.90 (0.07)	0.98 (0.06)	< 0.001
BMI (m^2^/kg)	22.94 (2.81)	26.46 (2.97)	< 0.001
SBP (mmHg)	117.03 (15.79)	124.18 (14.73)	< 0.001
DBP (mmHg)	74.79 (9.70)	77.49 (8.69)	0.007
TG (mg/dl)	109.72 (99.99)	188.90 (154.71)	< 0.001
Fasting glucose (mg/dl)	109.60 (55.14)	140.54 (54.15)	< 0.001
HDL (mg/dl)	49.05 (11.45)	37.68 (7.80)	< 0.001
MetS score			
0	68 (31.9)		< 0.001
1	68 (31.9)		
2	77 (36.2)		
3		62 (45.6)	
4		52 (38.2)	
5		22 (16.2)	

Data are presented as *n* (%) for categorical and mean (standard deviation) for continuous variables. *USD* US dollars, *MET* metabolic equivalent, *WHR* waist-hip ratio, *BMI* body mass index, *SBP* systolic blood pressure, *DBP* diastolic blood pressure, *TG* triglyceride, *HDL* high-density lipoprotein; for MetS score: number of components that meet the NCEP-ATP III criteria

**Table 2:** Differences of DNA methylation age between MetS and control groups

	Control	MetS	*p* value
Age, years	59.74 (8.21)	62.25 (8.49)	< 0.001
DNAmAge (years)			
Pan-tissue	62.99 (7.62)	62.05 (7.53)	0.262
Hannum	66.46 (7.60)	67.49 (7.21)	0.204
PhenoAge	56.02 (8.30)	56.50 (7.51)	0.569
GrimAge	65.05 (7.16)	67.21 (6.52)	0.004
SEM	2.89 (0.02)	2.88 (0.02)	0.710
DNAm-based Protein surrogates			
DNAm ADM	347.94 (19.71)	353.19 (22.35)	0.026
DNAm B2M	1695400.53 (118063.24)	17164680.23 (111301.02)	0.093
DNAm CystatinC	657922.87 (30767.76)	663421.07 (33629.34)	0.125
DNAm GDF15	778.64 (114.38)	792.13 (110.96)	0.275
DNAm Leptin	6425.81 (3842.37)	6812.72 (3994.74)	0.371
DNAm PAI1	18346.47 (3169.11)	19880.95 (2926.86)	< 0.001
DNAm TIMP1	34309.52 (1239.02)	34686.02 (1147.70)	0.004
DNAm Packyrs	23.57 (12.70)	23.88 (12.05)	0.813

Data are presented mean (standard deviation) using Student’s *t* test or Mann–Whitney *U* test.* SEM*, stochastic epigenetic mutation; *DNAm ADM* adrenomedulin, *DNAm B2M* beta-2-microglobulin, *DNAm cystatin C* cystatin C, *DNAM GDF15* growth differentiation factor 15, *DNAm leptin* leptin, *DNAm PAI1* plasminogen activation inhibitor 1, *DNAm TIMP1* tissue inhibitor metalloproteinase

### Association between chronological age and epigenetic ages in metabolic syndrome state

**Fig. 1:**
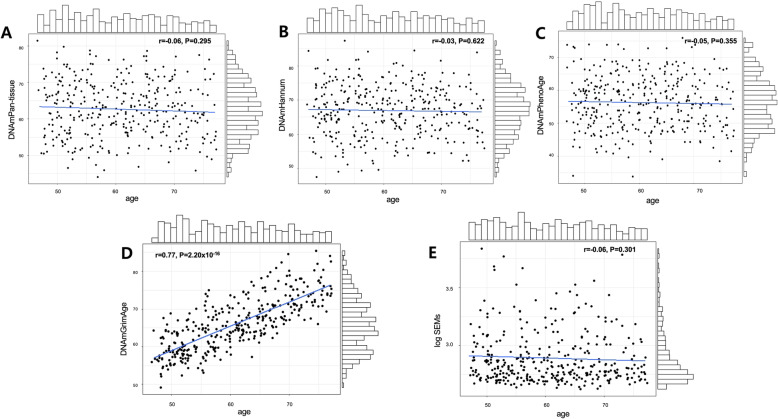
Correlation of five DNAm epigenetic clocks with chronological age. Scatter plots of DNA methylation age vs. chronological age. **a** Pan-tissue. **b** Hannum. **c** PhenoAge. **d** GrimAge. **e** Stochastic epigenetic mutations

The correlation between chronological age and the selected four epigenetic clocks are shown in Fig. 1. DNAm GrimAge strongly correlated with chronological age (*r* = 0.77, *p* < 0.001). while the other three clocks, Pan-tissue, Hannum, PhenoAge, and SEMs showed no correlation with chronological age (*P* > 0.050). Correlation was also observed between DNAm GrimAge and chronological age, when stratified by sex, regional area, age group, and smoking status (*r* = 0.43–0.78, Fig. S1). Moreover, DNAm-based surrogate protein markers including GDF15, CysteinC, B2M, and TIMP1, also correlated with chronological age (*p* < 0.010, Fig. S2).

**Fig. 2:**
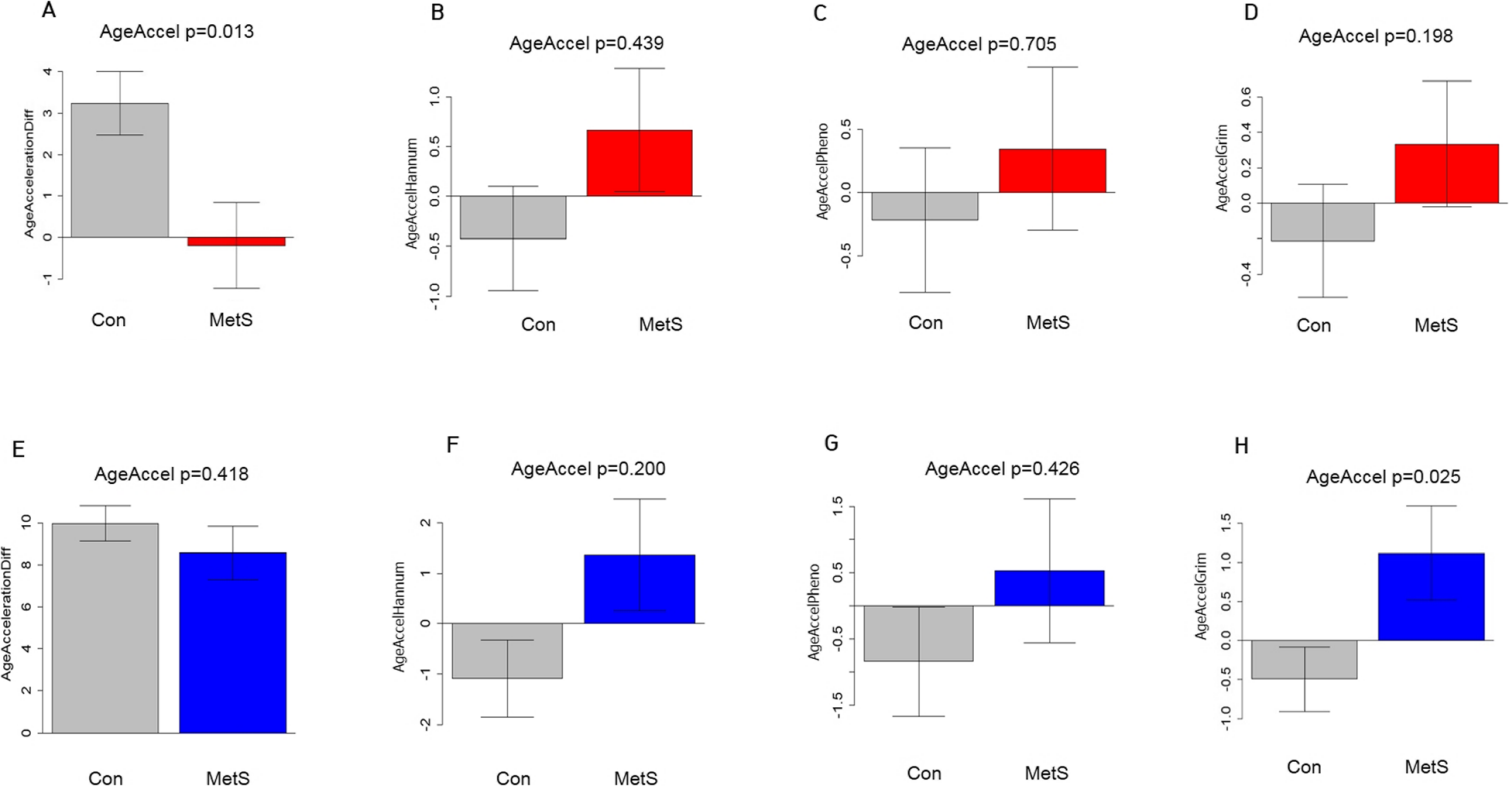
DNAm age acceleration levels, and the number of stochastic epigenetic mutations between MetS cases and controls, in all subjects (**a**–**e**, upper figures) and the middle-age group (**f**–**j**, lower figures). **a**, **f** Pan-tissue acceleration. **b**, **g** Hannum’s acceleration. **c**, **h** PhenoAge acceleration. **d**, **i** GrimAge acceleration. **e**, **j** SEMs

Positive values of accelerated DNAmAge indicated faster biological aging, based on chronological age, while negative values indicated decelerated aging. Accelerated GrimAge, in the peripheral blood, differed between the MetS and control groups (Fig. 2). In particular, positive DNAm GrimAge, from MetS cases, was observed in the middle-age group (Fig. 2h, *p =* 0.025). In contrast, Pan-tissueAge, HannumAge, PhenoAge, and SEMs, in blood did not differ between MetS and control groups. We also investigated whether MetS associated with age-adjusted plasma protein markers. As a result, DNAmPAI1 levels were significantly higher in MetS cases than in controls (Fig. S3(A), *P* < 2.40 × 10^−5^). These MetS status assays associated with increases of DNAm GrimAge in the middle-age group.

### Accelerated DNAm GrimAge associates with MetS and its scores

We evaluated whether accelerated DNAm GrimAge associates with the incidence of MetS (Table 3). Every one-year gain of GrimAge associated with a 16% increase risk of MetS only in middle-age group (OR = 1.16, 95% CI = [1.01, 1.35]; *p* = 0.046). In addition, we observed the association of GrimAge with an increasing risk of MetS, in females (OR = 1.15, 95% CI = [1.01, 1.33]; *p* = 0.047), non-smokers (OR = 1.2, 95% CI = [1.05, 1.37]; *p* = 0.007), and never-drinkers (OR = 1.19, 95% CI = [1.02, 1.39]; *p* = 0.021). In MetS components, fasting glucose levels associated with accelerated DNAm GrimAge (estimate (S.E), 0.01 (0.005); *p* = 0.045, Table 4). In estimators of aging, increased DNAmPAI1AdjAge (age-adjusted estimate of DNAmPAI1) associated with all MetS components, except for hypertension (Table 4). It seems that age-adjusted DNAm PAI1 outperforms accelerated GrimAge, for several MetS components. These findings demonstrate that the DNAm GrimAge indication estimator is quite accurate in predicting both chronological age, and biological age in MetS.

**Table 3:** Odds ratios for metabolic syndrome for accelerated GrimAge and age-adjusted DNAmPAI1

	AgeAccelGrim	*p* value	DNAmPAI1AdjAge	*p* value
Age group				
Middle-aged (< 60 years)	1.16 [1.1, 1.35]	0.046	1.0003[1.0001, 1.0004]	< 0.001
Elderly (≥ 60 years)	1.07 [0.94, 1.23]	0.311	1.0001 [1, 1.0002]	0.023
Living area				
Rural	1.05 [0.93, 1.19]	0.465	1.0001 [1, 1.0002]	0.047
Urban	1.06 [0.98, 1.15]	0.123	1.0004 [1.0002, 1.0005]	< 0.001
Sex				
Female	1.15 [1, 1.33]	0.047	1.0001 [1, 1.0003]	0.007
Male	1.06 [0.92, 1.21]	0.446	1.0002 [1.0001, 1.0004]	< 0.001
Smoking				
Never smoker	1.2 [1.05, 1.37]	0.007	1.0002 [1.0001, 1.0003]	< 0.001
Former smoker	0.9 [0.71, 1.14]	0.384	1.0001 [0.9999, 1.0004]	0.210
Current smoker	1.21 [0.95, 1.54]	0.123	1.0004 [1.0002, 1.0007]	< 0.001
Alcohol drinking				
Never drinker	1.19 [1.02, 1.39]	0.021	1.0001 [1, 1.0002]	0.020
Regular drinker	1.03 [0.9, 1.19]	0.655	1.0002 [1.0001, 1.0004]	< 0.001

Data are presented as Odds ratio [95% confidence interval] of metabolic syndrome. *AceAccelGrim*, accelerated DNAm GrimAge; *DNAmPAIAdjAge*, age-adjusted DANmPAI1; chronological age, sex, regional area and DNAmPACKYRS were included in the multiple linear regression

**Table 4:** Associations between age acceleration and lifestyle factors in middle-age group

	AgeAccelGrim		DNAmPAI1AdjAge	
Factors	Estimate (S.E)	*p* value	Estimate (S.E)	*p* value
Fasting glucose (mg/dl)	2.31(1.15)	0.045	15.49(3.71)	<0.001
HDL (mg/dl)	-0.28(0.24)	0.248	-39.44(18.58)	0.035
Waist circumference (cm)	0.05(0.16)	0.734	75.98(28.48)	0.008
Triglyceride (mg/dl)	-1.15(3.23)	0.722	0.98(1.40)	0.488
SBP (mmHg)	0.12(0.25)	0.622	32.70(17.79)	0.070
DBP (mmHg)	0.13(0.16)	0.432	49.45(28.19)	0.081
Abdominal fat (%)	0.0009(0.0008)	0.261	12133.46(5445.95)	0.027
Body fat (kg)	0.05(0.10)	0.594	87.27(45.71)	0.058
Body fat (%)	0.08(0.10)	0.395	85.12(47.94)	0.078
BMI (m^2^/kg)	0.04(0.06)	0.482	182.58(77.65)	0.020

Data are presented as estimates [standard error]. Estimate, difference in factor per year of epigenetic age acceleration. Chronological age, sex, and regional area were included in multiple linear regression. *AgeAccelGrim* DNAm GrimAge Acceleration, *DNAmPAI1AdjAge* age-adjusted DNAmPAI1, *HDL* high-density lipoprotein, *SBP* systolic blood pressure, *DBP* diastolic blood pressure, *BMI* body mass index

**Fig. 3:**
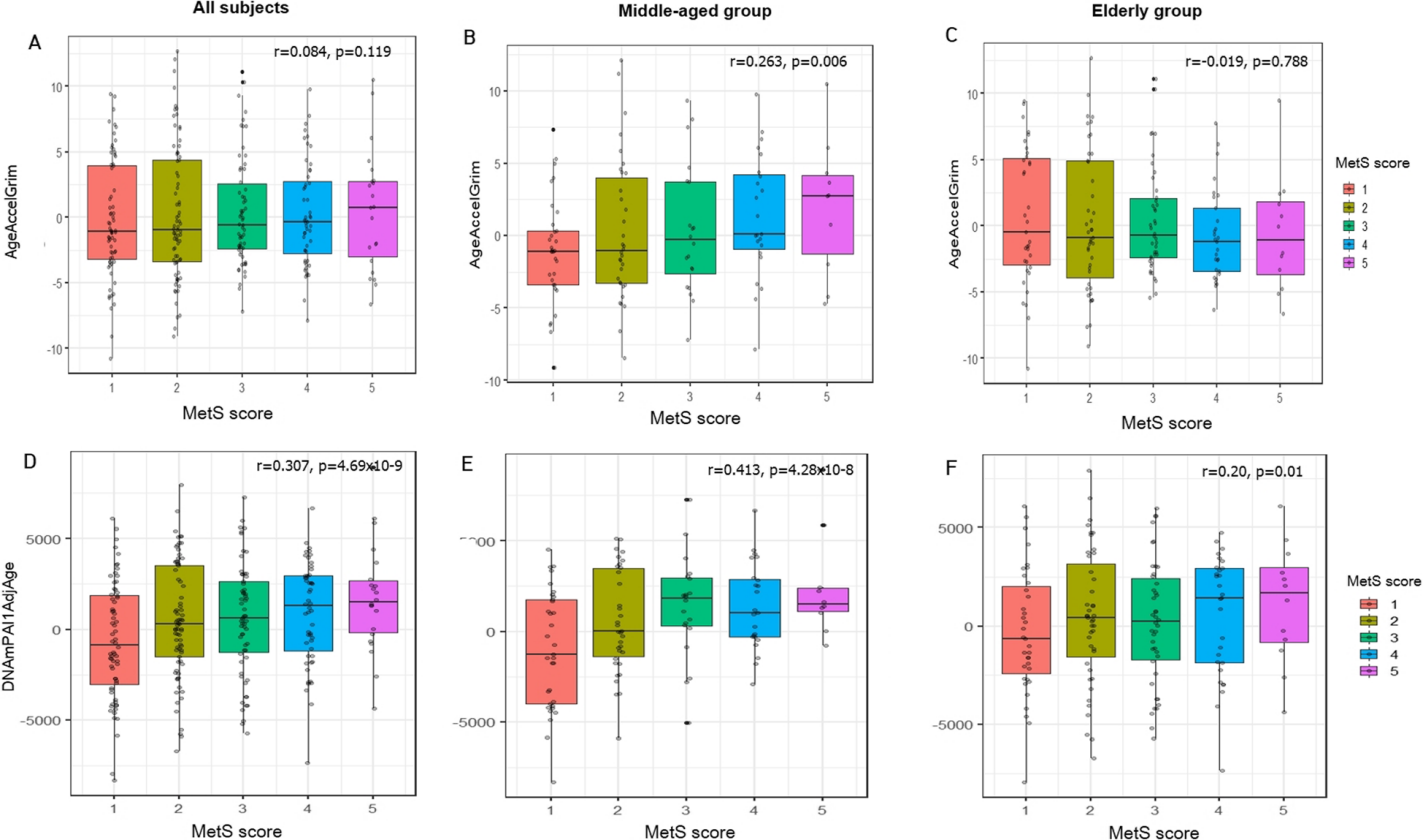
Box-plots of measured accelerated DNA GrimAge, or age-adjusted DNAmPAI1 by MetS scores. Centerline, median; box limits, upper and lower quartile; whiskers, 1.5 × interquartile range; and points, outliers. Age acceleration was adjusted by regional area, BMI, sex, smoking status, and chronological age. **a**, **d** All subjects. **b**, **e** Middle-age group. **c**, **f** Elderly group

We further identified an association between accelerated GrimAge and MetS scores. Figure 3 shows the result of correlating MetS score, and accelerated GrimAge, in all participants, as well as results from the middle-age and elderly groups. Positive correlation between accelerated GrimAge and MetS scores was also observed in the middle-age group (*r* = 0.26, *p =* 0.006; Fig. 3b). However, there was no significant association between accelerated GrimAge in the elderly age group (*r* = − 0.02, *p* = 0.788; Fig. 3c), although the effect of age-adjusted PAI1, on MetS scores, was observed in all groups (Fig. 3d, f).

## Discussion

This is the first study to apply epigenetic clocks, with MetS, to a Korean population. We found that DNAm GrimAge strongly correlated with chronological age (*r* = 0.77, *P* < 0.001). However, three other well-known epigenetic clocks (Pan-tissue, Hannum and PhenoAge), and SEMs, showed no association (*P* > 0.050). The Pan-tissue clock used a DNA methylation profile of 353 CpGs, without adjustment from 51 multiple tissues, from 8000 samples, to reflect the intrinsic aging process [8]. HannumAge was estimated using 71 CpG sites from whole blood from 656 individuals, as associated with the risk of all-cause mortality, and related covariates such as gender, BMI, diabetes, ethnicity, and batch [7]. While both the Pan-tissue and HannumAge clocks were estimated by penalized linear regression models, only 6 CpG sites were shared between the two clocks. The PhenoAge clock [9] was developed using penalized regression in which the hazard of age-related mortality was regressed on nine clinical variables (including biochemical properties, tissue function, immune function), and chronological age. This clock is based on methylation profiles of 513 CpGs from the third National Health and Nutrition Examination Survey (*N* = 9926), and InCHIANTI (*N* = 456) data, using whole blood.

Four previous studies investigated DNAmAges and MetS [10, 13–15]. Although previous MetS studies reported that intrinsic and extrinsic epigenetic age acceleration (IEAA and EEAA) positively associated with positive MetS status [13, 14], we could not replicate those results (Supplementary Table 2). These study populations were mainly based on Caucasians, African Americans, and Blacks, ethnicities different from a Korean population. For example, Quach et al. reported IEAA and EEAA associated with MetS, including the study participants composed of only 3% of Asian or Pacific Islanders [14]. Another study investigated the association between the Hannum clock and MetS, based on U.S. military veterans, including Whites and Hispanics, using the Infinium EPIC DNA methylation chip [15]. Lu et al., the creators of DNAmGrimAge, associated that algorithm with MetS, based on a multiethnic group of Caucasian, African, and Hispanic populations [10]. Horvath et al. previously reported that epigenetic aging rate significantly correlates with sex and ethnicity [16]. Since our result is based on a Korean population, this may affect its discordance from results of previous studies using DNAmAges, and our results were also likely influenced by differences in study sample sizes and the source of DNA.

The most interesting finding is that the risk of MetS associated with increased accelerated DNAm GrimAge, and age-adjusted DNAm PAI1, in the middle-age group, but not in the elderly group. In particular, MetS scores positively associated with accelerated DNAm GrimAge, indicating greater accelerated epigenetic aging, depending on the number MetS components, in the middle-age group. The peak age for MetS was reported to be under 50, while the prevalence decreased with aging, in Korea [17].

DNA methylation age, in blood, can predict the age of onset of chronic diseases [18]. Similarly, the change of DNAm age is faster in children, due to developmental growth [8, 19], and degenerative phenotypes, such as body mass index (BMI), which accelerate epigenetic clocks in blood, but only in the middle-age group (age, 40–49 years), in Finnish [20]. Research is now increasingly showing that the origins of risk for chronic conditions, such as diabetes and heart disease, begin in early childhood, or even earlier [21, 22]. Thus, smoking, lack of physical activity, inadequate diet, and other established adult risk factors, might put individuals at relatively greater risk of developing chronic diseases, at older ages.

A DNAm-based plasma protein, DNAm PAI1, and its age-adjusted one, could represent accurate biomarkers of ageing and MetS state. DNAm GrimAge was developed based on 7 DNAm estimators, including DNAm PAI1, and DNAm PACKYEAR. DNAm PAI1 and age-adjusted PAI1 were reported to be involved in lung function [23] and type 2 diabetes [10]. In addition, DNAm PAI1 was shown to outperform DNAm GrimAge, in several chronic diseases [10, 23]. We also found that MetS components, and obesity-related factors such as BMI, significantly associate with age-adjusted DNAm PAI1 (Table 4, *P* < 0.02), even though the results were not consistent with DNAm GrimAge. Therefore, further study of DNAm-related protein estimators is an important step for predicting chronological and biological ageing.

There are some limitations to the study. As mentioned in the introduction, one theory of aging involves oxidative stress, which is connected to biological aging; therefore, diet and nutrients are attributed to pro-oxidative or anti-oxidative factors. Lack of information for dietary patterns might also limit estimates of biological age. Besides, due to a limited number of individuals included in our study, our result should be further confirmed and strengthened by other validation studies using larger cohorts.

## Conclusions

In conclusion, we provide evidence that accelerated DNAm GrimAge, and plasma protein levels, associated with MetS, in a Korean population. In addition, this finding was significant in a middle-age group (< 60 years). However, we could not observe that other epigenetic clocks were involved in MetS progression in our study population. While further comparison, with different populations, is required, we suggest that epigenetic clocks, such as DNAm GrimAge, could be a useful biomarker for the diagnosis of metabolic disease, and establish the role of epigenetic aging processes, in such diseases.

## Methods

### Study population

The present study was approved by the institutional review board of Seoul National University (E1908/001-004). Participants were from the Korean Genome Epidemiology Study (KoGES), which is now on its fifth 2-year follow-up phase, in 2011–2012 (Ansan-Ansung community-based cohort study). Its study design, sampling, concept, and consent are described in a previous study [24]. Study samples were drawn from the Korea Association REsource (KARE) for which data on DNA methylation was available. Educational attainment was categorized into three groups: less than 7 total years (elementary school graduates), 7–9 years (middle school graduates), and more than 10 years (high school graduates). Monthly household income was also categorized into three groups: less than $1000 USD (in 2014), $1000–$2000, and ≥ $2000. Physical activity was quantified by metabolic equivalent (MET) intensity [25]. Obesity was diagnosed using different methods: body fat and abdominal fat were assessed by multi-frequency bioelectrical impedance analysis (MF-BIA; InBody 3.0, Biospace, Seoul, Korea). Abdominal fat (cm^2^) was measured using dial-energy X-ray absorptionmetry (DXA). We also investigated waist circumference (cm), hip (cm), waist-hip ratio, and BMI (cm/m^2^) for assessments of obesity [26]. Epidemiological and biochemical data for this study was provided by the National Biobank of Korea, and KoGES, according to the approval of the sample and data access committee.

### DNA methylation profiling

Genomic DNA from peripheral blood was used for DNA methylation assessment for this study. High-quality genomic DNA (500 ng for each sample) was modified by sodium bisulfate, using an EZ DNA methylation kit (Zymo Research, Orange, CA, USA) according to the manufacturer’s instruction. Genome-wide DNA methylation was profiled using the Illumina Infinium HumanMethylation450 BeadChip (Illumina, San Diego, CA, USA), composed of > 485,000 CpGs, and covering 99% of RefSeq genes. Hybidized DNA was scanned using an Illumina iScan. GenomeStudio V2011 (Methylation Module, R 2.11) software was used for quantification and image analysis of the methylation data (Illumina). All samples passed GenomeStudio quality control steps, based on built-in control probes for staining, hybridization, extension, and specificity, and the bisulfite conversion efficiency was high (intensity signal > 4000). We followed the quality control procedure using the Bioconductor minfi package [27]. For example, data was filtered to remove CpGs with high detection *p* values. The procedure for this DNA methylation assessment has been described previously [28].

### Metabolic syndrome (MetS) definition

MetS is characterized by the combination of several components, including abdominal obesity, hypertension, dyslipidemia, insulin resistance, and glucose intolerance, important precursors of cardiovascular disease and type 2 diabetes. Of these, MetS is defined by the presence of three or more of the following five components, according to the NCEP-ATP III criteria, except for the determination of central obesity [29]. Waist circumference cut-off value was based on the report by the Korean Society for the Study of Obesity: (1) central obesity, given as waist-high circumference (≥ 90 cm for men and ≥85 cm for women); (2) high concentrations of serum triglyceride (≥ 150 mg/dL); (3) low concentrations of serum high-density lipoprotein cholesterol (< 40 mg/dL for men and < 50 mg/dL for women); (4) hypertension (systolic/diastolic pressure ≥ 130/85 mmHg), or taking antihypertensive medications; and (5) high concentrations of fasting glucose (≥ 100 mg/dL) or taking antidiabetes medications. MetS score was calculated for each subject, as the summation of the number above the cut-off, for each MetS component, ranging from 0 to 5.

### DNA methylation age and stochastic epigenetic mutation calculation

DNA methylation ages (DNAmAge) were calculated using an online age calculator (http://dnamage,genetics.ucla.edu/) developed by Horvath (5), with the normalization feature set to “true.” We calculated 4 DNAmAge (Pan-tissue, Hannum, PhenAge, GrimAge) and GrimAge surrogates, including seven proteins: adrenomedulin, beta-2-microglobulin, cystatin C, growth differentiation factor 15, leptin, PAI1, and tissue inhibitor metalloproteinaise, and DNAm PACKYEAR. We identified SEMs using the procedure described by Gentilini et al. [11]. Briefly, for each CpG, considering the distribution of DNAm beta values, across all samples, we computed the interquartile range (IQR, the difference between the third quartile (Q3) and the first quartile (Q1)), and defined SEMs at a methylation value lower than Q1-(3 × IQR), or greater than Q3 + (3 × IQR). Finally, for each participant, we calculated the total number of SEMs across all CpGs and analyzed them using a logarithmic scale.

### Statistical analyses

For the characterization of subjects, data were presented as means (standard deviations), for continuous variables, or as percentages (%), for categorical variables (Table 1). Prior to analysis, all variables available were examined for departure from normality, and log-transformation was made for the skewed distribution with a long right tail. Distinctions between different groups were detected using the Mann–Whitney *U* test, for non-normally distributed continuous variables, and the chi-square test, for categorical variables. Pearson correlation coefficients were used to test for correlation between chronological age and four different DNAmAges, and between chronological age and SEMs and between chronological age and age-adjusted DNAm plasma protein levels, and between DNAmAge acceleration and MetS scores. Student’s *t* test or Mann–Whitney *U* test was performed for comparisons between MetS and control groups for accelerated DNAmAge. We also performed linear regression analysis between DNAmAge and MetS scores, and between epigenetic age acceleration and MetS score adjusted by chronological age, sex, regional area, smoking status, and BMI. Logistic regression was used to test the association between risk of MetS and accelerated GrimAge, with covariates including chronological age, sex, region, and DNAm PACKYEAR (Table 3). For accelerated GrimAge and age-adjusted DNA PAI1, effects on lifestyle and obesity-related factors, multiple regression models were fitted using potential confounding factors, such as chronological age, sex, and region (Table 4). *p* values < 0.05 were considered statistically significant. All statistical analyses were performed using R Software (version 2.14.0; R Foundation for Statistical Computing, Vienna, Austria)

## Supplementary information

**Additional file 1: Table S1.** Difference of DNAm age and DNAm-based protein estimators between MetS and control groups. Table S2. Differences of internal and external DNAm age accelerations between MetS and control groups. Table S3. MetS components and obesity-related factors between middle-aged and elderly groups.

**Additional file 2: Figure S1.** Association between DNAm GrimAge and chronological age stratified by age group, sex, smoking status and regional area. (all p <0.05 for correlations, A~D). Figure S2. Correlation between DNA methylation ages including protein based age estimators. Figure S3. DNAm age acceleration levels between controls and MetS cases in all subjects (A~E, upper figures) and middle-aged group (F~J, lower figures).

## Data Availability

DNA methylation dataset and epidemiological data for KARE project are third party data and are available under the approval of the data access committee of the National Biobank of Korea (http://www.nih.go.kr/NIH/eng/contents/NihEngContentView.jsp?cid=65714&menuIds=HOME004-MNU2210-MNU2327-MNU2329-MNU2338).

## Abbreviations

BMI: Body mass index
DNAm: DNA methylation
DNAmAge: DNA methylation ages
DNAmPAI1AdjAge: Age-adjusted estimate of DNAmPAI1
EEAA: Extrinsic epigenetic age acceleration
IEAA: Intrinsic epigenetic age acceleration
KoGES: Korean Genome Epidemiology Study
MET: Metabolic equivalent
MetS: Metabolic syndrome
PAI1: Plasminogen activator inhibitor-1
SEMs: Stochastic epigenetic mutations
