# Supplementary material for: The influences of DNA methylation and epigenetic clocks, on metabolic disease, in middle-aged Koreans
Source: Clin Epigenetics. 2020 Oct 15;12:148. doi: 10.1186/s13148-020-00936-z (PMC7558749; doi:10.1186/s13148-020-00936-z)
Supplement: Supplementary file 1 — Additional file 1: Table S1. Difference of DNAm age and DNAm-based protein estimators between MetS and control groups. Table S2. Differences of internal and external DNAm age accelerations between MetS and control groups. Table S3. MetS components and obesity-related factors between middle-aged and elderly groups. [file 13148_2020_936_MOESM1_ESM.docx]

Table S1. Difference of DNAm age and DNAm-based protein estimators between MetS and control groups.

|  | middle-aged group  (<60 years, n=163) | Eldery group  (≥60 years, n=186) | *P value* |
| --- | --- | --- | --- |
| Female | 70 (42.94) | 102(54.83) | 0.03 |
| Age (year) | 53.03 (3.87) | 67.46(5.01) | <0.01 |
| DNAmAge |  |  |  |
| Pan-tissue (year) | 62.55 (8.07) | 62.68 (7.17) |  |
| Hannum (year) | 66.75 (8.07) | 66.95 (6.89) | 0.20 |
| Phenoage (year) | 56.04 (8.39) | 56.35 (7.64) | 0.57 |
| GrimAge (year) | 60.98 (4.87) | 70.20 (5.59) | <0.01 |
| ADM | 342.27(20.32) | 356.75(19.03) | 0.03 |
| B2M | 1618345.35 (79328.29) | 1778340.49 (87644.18) | 0.09 |
| CystatinC | 639578.67 (23710.74) | 678018.90 (27156.66) | 0.12 |
| GDF15 | 721.31 (89.24) | 838.74 (103.00) | 0.27 |
| Leptin | 6599.60 (4084.18) | 6556.42 (3744.75) | 0.37 |
| Packyrs | 22.79 (12.53) | 24.47(12.33) | 0.81 |
| PAI1 | 18655.99 (3090.71) | 19197.21 (3211.59) | <0.01 |
| TIMP1 | 33450.15 (641.47) | 35337.92 (866.19) | <0.01 |
| Telomere length | 6.98 (0.22) | 6.94 (0.20) | 0.21 |

Table S2. Differences of internal and external DNAm age accelerations between MetS and control groups.

| DNAmAge acceleration | Control (n=213) | MetS (n=136) | *P value* |
| --- | --- | --- | --- |
| IEAA | 0.23 (6.74) | -0.35 (6.50) | 0.42 |
| EEAA | -0.42 (9.25) | 0.66 (8.49) | 0.26 |

Values are presented as means(standard deviations). IEAA, intrinsic epigenetic age acceleration; EEAA, extrinsic epigenetic age acceleration

Table S3. MetS components and obesity-related factors between middle-aged and elderly groups.

|  | Middle-aged group  (<60 years, n=163) | Eldery group  (≥60 years, n=186) | *P value* |
| --- | --- | --- | --- |
| SBP (mmHg) | 116.84 (13.95) | 122.42 (16.80) | <0.01 |
| DBP (mmHg) | 77.18 (9.38) | 74.67 (9.29) | 0.02 |
| Triglyceride | 150.71(176.33) | 131.69 (65.89) | 0.64 |
| Fasting glucose | 127.05 (62.77) | 116.92 (50.55) | 0.20 |
| HDL | 46.03 (13.06) | 43.38 (10.00) | 0.03 |
| BMI (m^2^/kg) | 24.71 (3.15) | 23.97 (3.48) | 0.05 |
| Waist circumference (cm) | 82.96 (9.61) | 86.57 (8.87) | <0.01 |
| Hip circumference (cm) | 91.67 (5.69) | 90.83 (5.72) | 0.17 |
| Abdominal fat (cm) | 74.92 (39.16) | 87.69 (42.14) | 0.14 |
| Abdominal fat (%) | 0.90 (0.05) | 0.93 (0.05) | <0.01 |
| Bodyfat (%) | 24.96 (7.24) | 26.72 (7.72) | 0.04 |
| Bodyfat (kg) | 16.40 (5.79) | 16.14 (5.97) | 0.72 |
