# Supplementary figures and images for: The influences of DNA methylation and epigenetic clocks, on metabolic disease, in middle-aged Koreans
Source: Clin Epigenetics. 2020 Oct 15;12:148. doi: 10.1186/s13148-020-00936-z (PMC7558749; doi:10.1186/s13148-020-00936-z)

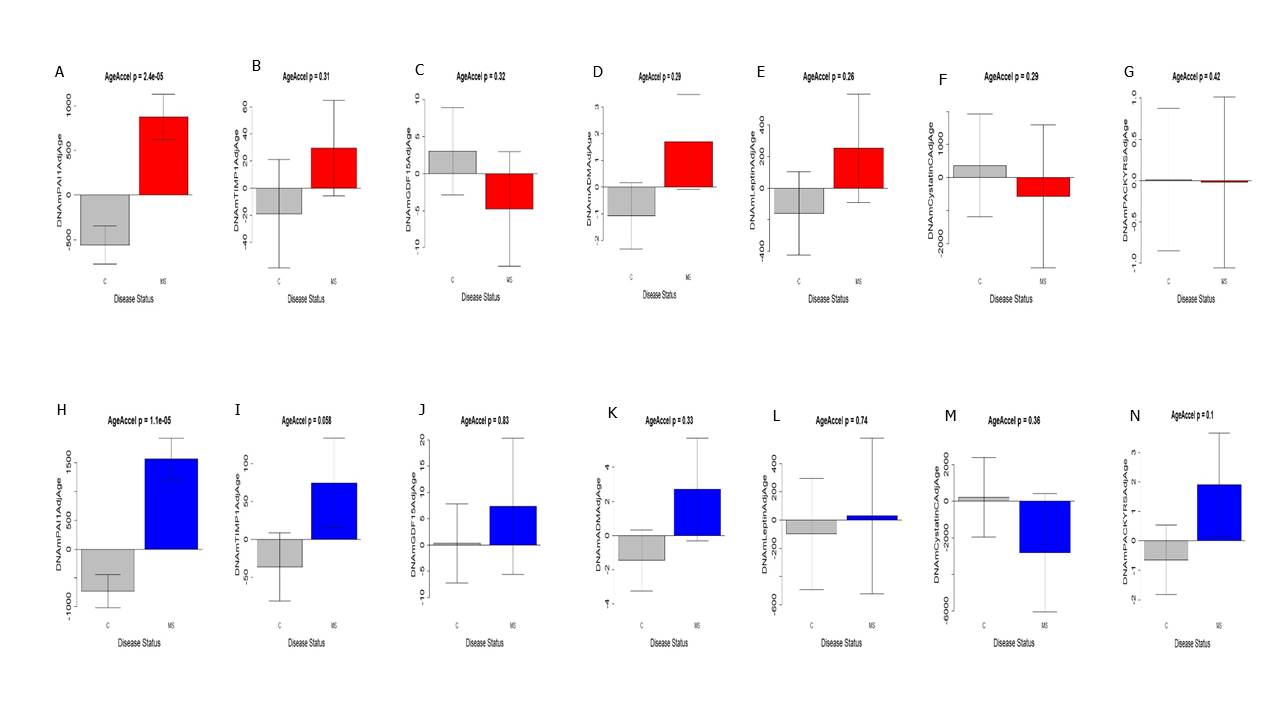

Supplement: Supplementary file 2 — Additional file 2: Figure S1. Association between DNAm GrimAge and chronological age stratified by age group, sex, smoking status and regional area. (all p <0.05 for correlations, A~D). Figure S2. Correlation between DNA methylation ages including protein based age estimators. Figure S3. DNAm age acceleration levels between controls and MetS cases in all subjects (A~E, upper figures) and middle-aged group (F~J, lower figures). [file 13148_2020_936_MOESM2_ESM.jpg]
